# The effect of depressive symptoms on disability-free survival in healthy older adults: A prospective cohort study

**DOI:** 10.1111/acps.13513

**Published:** 2022-11-11

**Authors:** Greg Roebuck, Mojtaba Lotfaliany, Bruno Agustini, Malcolm Forbes, Mohammadreza Mohebbi, John McNeil, Robyn L. Woods, Christopher M. Reid, Mark R. Nelson, Raj C. Shah, Joanne Ryan, Anne B. Newman, Alice Owen, Rosanne Freak-Poli, Nigel Stocks, Michael Berk, ASPREE Investigator Group

**Affiliations:** 1 The Institute for Mental and Physical Health and Clinical Translation (IMPACT), School of Medicine, Deakin University, and Barwon Health, Geelong, Victoria, Australia; 2 Phoenix Australia – Centre for Posttraumatic Mental Health, Department of Psychiatry, University of Melbourne, Parkville, Victoria, Australia; 3 Department of Psychiatry, University of Melbourne, Parkville, Victoria, Australia; 4 Biostatistics Unit, Deakin University, Geelong, Victoria, Australia; 5 School of Public Health and Preventive Medicine, Monash University, Melbourne, Victoria, Australia; 6 School of Population Health, Curtin University, Perth, Western Australia, Australia; 7 Menzies Institute for Medical Research, University of Tasmania, Hobart, Tasmania, Australia; 8 Department of Family Medicine and Rush Alzheimer’s Disease Center, Rush University Medical Center, Chicago, Illinois, USA; 9 School of Public Health, University of Pittsburgh, Pittsburgh, Pennsylvania, USA; 10 Discipline of General Practice, Faculty of Health and Medical Sciences, University of Adelaide, Adelaide, South Australia, Australia; 11 Orygen, the National Centre of Excellence in Youth Health, and the Florey Institute for Neuroscience and Mental Health, Department of Psychiatry, University of Melbourne, Melbourne, Victoria, Australia

**Keywords:** ageing, disability-free survival, healthspan, late-life depression, psychiatry

## Abstract

**Background:**

Gerontology and ageing research are increasingly focussing on healthy life span (healthspan), the period of life lived free of serious disease and disability. Late-life depression (LLD) is believed to impact adversely on physical health. However, no studies have examined its effect on healthspan. This study investigated the effect of LLD and subthreshold depression on disability-free survival, a widely accepted measure of healthspan.

**Methods:**

This prospective cohort study used data from the ASPirin in Reducing Events in the Elderly study. Participants were aged ≥70 years (or ≥65 years for African-American and Hispanic participants) and free of dementia, physical disability and cardiovascular disease. Depressive symptoms were measured using the 10-item Centre for Epidemiological Studies Depression Scale (CES-D-10). LLD and subthreshold depression were defined as CES-D-10 scores ≥8 and 3–7, respectively. Disability-free survival was defined as survival free of dementia and persistent physical disability.

**Results:**

A total of 19,110 participants were followed up for a maximum of 7.3 years. In female participants, LLD was associated with lower disability-free survival adjusting for sociodemographic and lifestyle factors, medical comorbidities, polypharmacy, physical function and antidepressant use (HR, 1.50; 95% CI, 1.23–1.82). In male participants, LLD was associated with lower disability-free survival adjusting for sociodemographic and lifestyle factors (HR, 1.30; 95% CI, 1.03–1.64). Subthreshold depression was also associated with lower disability-free survival in both sexes.

**Conclusions:**

LLD may be a common and important risk factor for shortened healthspan.

## 1 | INTRODUCTION

Life expectancy has increased dramatically since the mid-twentieth century. Between 1950 and 2017, global life expectancy at birth rose from 52.9 to 75.6 years for women and from 48.1 to 70.5 years for men.^1^ This ageing of the global population has been associated with a substantial increase in the prevalence of chronic diseases. In the developed world, 87% of people aged over 65 years suffer from at least one chronic illness and 66% suffer from two or more such illnesses.^2^ Given the suffering and disability caused by age-related chronic diseases, medicine is increasingly focussing on prolonging healthy life span, or “healthspan”. This is the period of life lived free of serious illness and disability.^3^ There is growing interest in the lifestyle and other factors that determine healthspan and the ability of clinical interventions to extend healthspan.^4–6^

Late-life depression (LLD) can be defined as major depressive disorder occurring in adults aged 60 years or older. It is common, with an estimated global prevalence of 13.3%.^7^ A substantial body of evidence suggests that LLD impacts adversely on older adults’ physical health. Longitudinal studies have found that it is associated with higher rates of all-cause and cardiovascular mortality.^8^ LLD also appears to be an independent risk factor for developing cardiovascular disease (CVD) and for poor outcomes in established CVD.^9^ Finally, LLD prospectively predicts the development of common geriatric syndromes, including frailty, dementia and disability.^10–12^

These findings suggest that LLD is likely to shorten healthspan. There is some evidence from studies not limited to geriatric populations that depression is associated with lower healthy life expectancy.^13,14^ A recent study of 9761 individuals aged 50 years and older in the United Kingdom found that the presence of depressive symptoms was associated with lower disability-free life expectancy across all age groups.^14^ Consulting a general practitioner about depressive symptoms for the first time after the age of 60 years also predicts shorter disability-free life expectancy.^15^ As far as we are aware, however, no large observational studies have investigated the effect of LLD, assessed using a validated measure of depressive symptoms, on healthspan.

The aim of the current study was to examine the effect of LLD and subthreshold depressive symptoms on disability-free survival in physically healthy older adults. Disability-free survival is a widely accepted measure of healthspan or health expectancy.^6,16^ In this study, it was defined as survival free of dementia and persistent physical disability. Persistent physical disability was defined in turn as self-reported severe difficulty or inability to perform independently one or more of six basic activities of daily life (ADLs) (walking across a room, bathing, dressing, transferring from a bed or chair, toileting and eating) for at least 6 months.^17^ We hypothesised that both LLD and subthreshold depression would be associated with lower disability-free survival in female and male participants. We further hypothesised that there would be a dose-response relationship, with LLD associated with a larger reduction than subthreshold depression. Secondarily, we aimed to explore the effects of LLD on the individual outcomes of all-cause mortality, incident dementia and incident persistent physical disability.

## 2 | MATERIALS AND METHODS

### 2.1 | Study population

This prospective cohort study used data from the ASPirin in Reducing Events in the Elderly (ASPREE) study, which was a large, multi-centre, population-based randomised controlled trial that investigated the effect of aspirin on disability-free survival and other outcomes in healthy older adults. Participants were recruited from Australia and the United States between 2010 and 2014. In Australia, recruitment was mostly based in general practise. In the United States, prospective participants were identified through clinic-based mailing lists, electronic medical records and responses to media advertisements. The inclusion criteria required participants to be aged 70 years or over (or 65 years or over for African-American and Hispanic participants in the United States) and able and willing to give informed consent. Exclusion criteria included a history of CVD or atrial fibrillation, a clinical diagnosis of dementia or score of <78 on the Modified Mini-Mental State Examination (3MS),^18^ physical disability, anaemia, a current or recurrent condition with a high risk of major bleeding, uncontrolled hypertension, absolute contraindication or allergy to aspirin, current continuous use of aspirin for secondary prevention, current continuous use of other antiplatelet or anticoagulant medication and any medical condition likely to cause death within 5 years. The design of the ASPREE study has been reported elsewhere.^4^

### 2.2 | Procedures and measures

Participants attended for assessments at baseline and then annually for the duration of their participation in the study. They also had telephone contact with the investigators every 3 months between visits. At each visit, participants were asked about demographic and lifestyle factors, their medical history and their medication use. They completed the LIFE disability questionnaire^19^ and other self-report measures. Their weight, height, waist circumference, blood pressure and heart rate were measured. A blood sample was taken to measure haemoglobin, creatinine, fasting glucose and fasting lipids and a urine sample was taken to measure urinary albumin:creatinine ratio. Every second year, hand grip strength was measured using a dynamometer and time to walk 3 m was measured.

### 2.3 | Measurement of depressive symptoms

Depressive symptoms were measured at baseline using the 10-item version of the Centre for Epidemiological Studies Depression Scale (CES-D-10).^20^ The CES-D-10 is a self-report questionnaire that assesses the severity of depressive symptoms over the previous week. It consists of 10 items rated on a 4-point Likert-type scale from 0 (“rarely or none of the time”) to 3 (“all of the time”). Using a cut-off score of ≥10, it has very tight agreement (*κ* = 0.97) with the full 20-item CES-D.^20^ A recent meta-analysis found that the 20-item CES-D has a sensitivity of 81% and a specificity of 78% for identifying LLD.^21^ LLD and subthreshold depression were defined as CES-D-10 scores of ≥8 and 3–7, respectively. Participants with a CES-D-10 score ≤2 were considered to have minimal or no depressive symptoms. The cut-off of ≥8 for LLD was consistent with previous studies using the CES-D-10.^22^ The ranges for the subthreshold depression and no depression groups were based on an earlier analysis by our group that showed that ASPREE participants’ CES-D-10 scores tended to follow four different trajectories: consistently low, consistently moderate, consistently high and initially low and then increasing scores.^23^ These ranges were derived from the interquartile ranges for the consistently moderate and consistently low classes in this analysis, respectively.

### 2.4 | Primary and secondary outcomes

Disability-free survival was defined as survival free of dementia or persistent physical disability and assessed as a composite of the first event of death, dementia or persistent physical disability. Dementia was defined according to the criteria in the fourth edition of the American Psychiatric Association’s *Diagnostic and Statistical Manual of Mental Disorders*^24^ (DSM-IV). Triggers for a dementia assessment included a 3MS score of <78, a decrease in 3MS score of >10.15 points from the participant’s baseline score after adjustment for age and level of education, a clinical diagnosis of dementia, a report of cognitive concerns to a specialist and the prescription of a cholinesterase inhibitor (for Australian participants). Dementia assessments involved additional cognitive and functional testing by research staff, the collection of ancillary data, including laboratory tests and neuroimaging, and a review of the participant’s medical records. The Dementia Adjudication Panel then determined whether the participant met DSM-IV criteria for dementia.^4^ The individual endpoints of all-cause mortality, incident dementia and incident persistent physical disability were treated as secondary endpoints. For participants who met the primary composite endpoint, follow up continued with respect to the remaining secondary endpoints.

### 2.5 | Statistical analysis

Differences in the baseline characteristics of the LLD, subthreshold depression and no depression groups were explored using a three-way analysis of variance for continuous variables and the chi-square test of independence for frequencies. For each endpoint, survival time was coded as the time between the baseline assessment and the occurrence of the endpoint or, if the endpoint did not occur, the final study visit. The Kaplan–Meier method was used to estimate survival functions for the three groups for each endpoint. The relationship between group and survival time was explored using Cox proportional hazards regression models. To explore the factors contributing to any associations between depressive symptoms and the endpoints, including mediators, moderators and confounding factors, five different regression models were tested. Each model adjusted for an additional set of covariates. Model 1 adjusted for sociodemographic factors (age and race). Model 2 further adjusted for lifestyle factors (body mass index [BMI], alcohol use history, smoking history, level of education and accommodation status). Model 3 further adjusted for the presence of common medical conditions (hypertension, dyslipidaemia, diabetes mellitus, chronic kidney disease (CKD), respiratory disease, gastroesophageal reflux disease (GORD), gout, Parkinson’s disease and a cancer history) and polypharmacy (simultaneous use of ≥5 medications). Model 4 further adjusted for measures of physical function (grip strength, gait speed and self-reported longest time walking without rest). Model 5 further adjusted for use of antidepressant medications at baseline. An *F*-test of overall significance was performed to calculate *p*-values for the three-way comparisons between the groups for each endpoint in Model 5. The threshold for statistical significance was set at 0.05 and the Benjamini-Hochberg procedure was used to adjust for multiple comparisons with the false discovery rate set to 0.05.^25^ Data for female and male participants were analysed separately.

## 3 | RESULTS

### 3.1 | Baseline characteristics of three groups and incidence of endpoints

A total of 19,110 participants completed the CES-D-10 at baseline and were included in the study: 10,799 female participants and 8311 male participants. Table 1 shows the sociodemographic and lifestyle characteristics, rates of common medical conditions and polypharmacy, performance on physical function measures, rates of antidepressant medication use and mean CES-D-10 scores at baseline for the LLD, subthreshold depression and no depression groups. Table 2 shows the incidences of the primary and secondary endpoints for the three groups during the follow-up period.

### 3.2 | Disability-free survival

**FIGURE 1:**
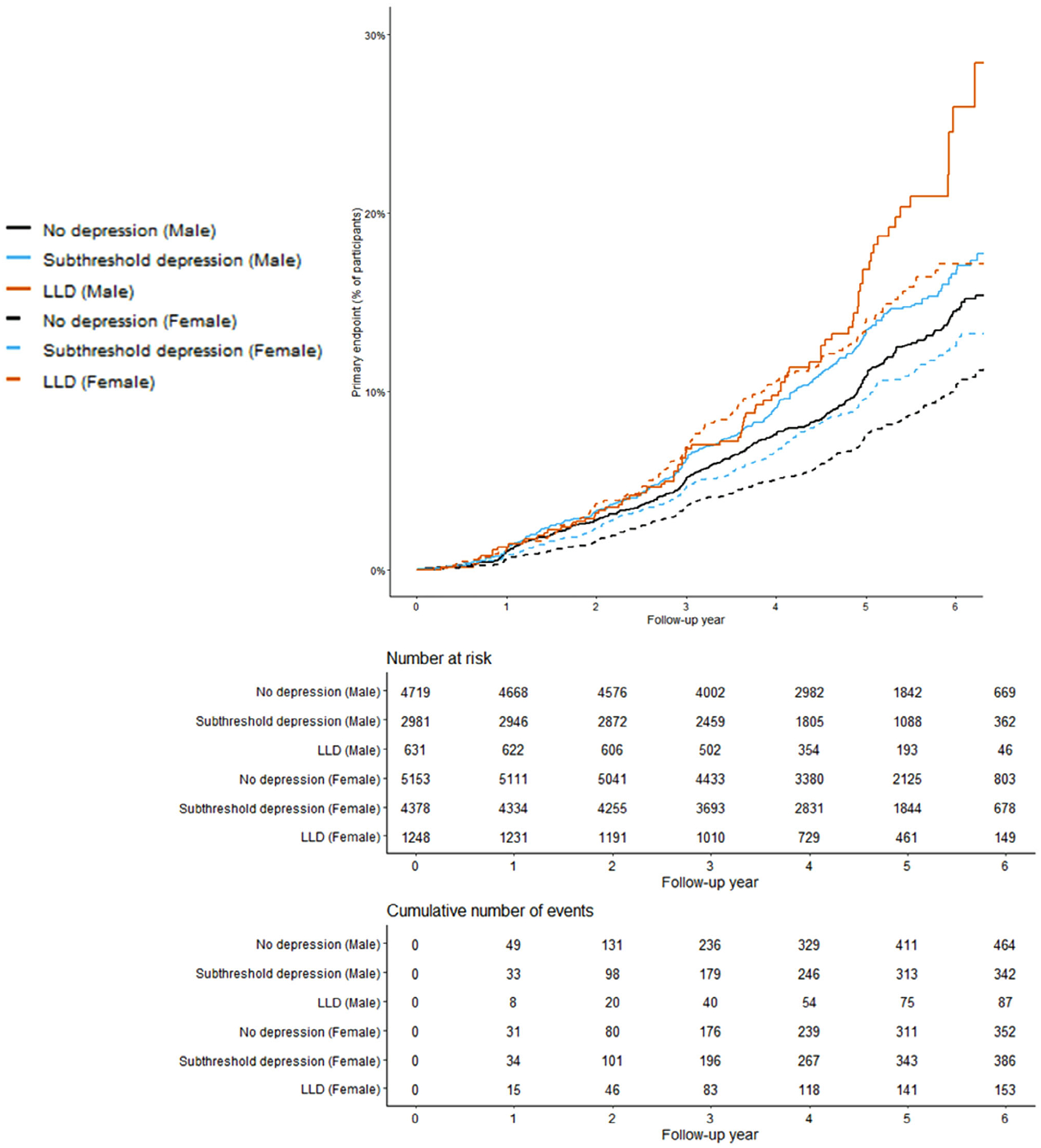
Cumulative incidence of the primary composite endpoint (defined as first event of death, dementia or persistent physical disability) for the three groups for female and male participants

After a median (range) follow-up period of 4.7 (0–7.3) years and 4.5 (0–7.3) years, 918 female participants and 917 male participants had died or developed dementia or persistent physical disability as a first event. Adjusting for all covariates, the LLD, subthreshold depression and no depression groups differed significantly for female participants (*p* < 0.001) but not male participants (*p* = 0.746) (Table 3 and Figure 1). For female participants, LLD was associated with lower disability-free survival adjusting for all covariates (hazard ratio (HR), 1.50; 95% confidence interval (CI), 1.23–1.82). Subthreshold depression was associated with lower disability-free survival adjusting for sociodemographic and lifestyle factors, medical comorbidities and polypharmacy (HR, 1.19; 95% CI, 1.03–1.38). The association became non-significant after adjustment was made for physical function, however. For male participants, LLD and subthreshold depression were associated with lower disability-free survival adjusting for sociodemographic and lifestyle factors (LLD: HR, 1.30; 95% CI, 1.03–1.64; subthreshold depression: HR, 1.30; 95% CI, 1.03–1.36). The associations became non-significant after adjustment was made for medical comorbidities and polypharmacy, however. The full results of the Cox proportional hazards regression analysis for Model 5, which included all covariates, are set out in Table S1 in the Supplementary Materials.

### 3.3 | All-cause mortality

After a median (range) follow-up period of 4.8 (0–7.3) years and 4.6 (0–7.3) years, 469 female participants and 583 male participants had died. Adjusting for all covariates, the three groups did not differ significantly for female participants (*p* = 0.029) or male participants (*p* = 0.501) when correction was made for multiple comparisons (Table 3 and Figure S1, Supplementary Materials). For female participants, LLD was associated with higher mortality adjusting for all covariates (HR, 1.44; 95% CI, 1.08–1.91). Subthreshold depression was associated with higher mortality adjusting for sociodemographic and lifestyle factors, medical comorbidities and polypharmacy (HR, 1.26; 95% CI, 1.03–1.55). The association became non-significant when adjustment was made for physical function, however. For male participants, subthreshold depression was associated with higher mortality adjusting for sociodemographic factors (HR, 1.19; 95% CI, 1.01–1.42). There was otherwise no association between depressive symptoms and mortality.

### 3.4 | Dementia

After a median (range) follow-up period of 4.6 (0–7.3) years and 4.5 (0–7.3) years, 302 female participants and 273 male participants had developed dementia. Adjusting for all covariates, the three groups did not differ significantly for female participants (*p* = 0.147) or male participants (*p* = 0.562) (Table 3 and Figure S2, Supplementary Materials). For female participants, LLD was associated with dementia adjusting for all covariates (HR, 1.43; 95% CI, 1.01–2.03). There were otherwise no associations between depressive symptoms and dementia for female or male participants.

### 3.5 | Persistent physical disability

After a median (range) follow-up period of 4.0 (0–7.0) years and 4.0 (0–7.0) years, 238 female participants and 174 male participants had developed persistent physical disability. Adjusting for all covariates, there was a significant difference between the three groups for female participants (*p* = 0.006) but not male participants (*p* = 0.045) following correction for multiple comparisons (Table 3 and Figure S3, Supplementary Materials). For female participants, LLD was associated with persistent physical disability adjusting for all covariates (HR, 1.87; 95% CI, 1.29–2.73). Subthreshold depression was associated with persistent physical disability adjusting for sociodemographic and lifestyle factors (HR, 1.35; 95% CI, 1.01–1.81). The association became non-significant when adjustment was made for medical comorbidities and polypharmacy. For male participants, LLD was associated with persistent physical disability adjusting for sociodemographic and lifestyle factors, medical comorbidities, polypharmacy and physical function (HR, 1.75; 95% CI, 1.06–2.90). The association became non-significant when adjustment was made for antidepressant medication use at baseline. Subthreshold depression was associated with persistent physical disability adjusting for all covariates (HR, 1.47; 95% CI, 1.05–2.06).

## 4 | DISCUSSION

To our knowledge, this is the first study to investigate the effect of LLD on disability-free survival. Consistent with our hypotheses, LLD was associated with lower disability-free survival in both sexes. In female participants, this association remained significant after adjustment for sociodemographic and lifestyle factors, medical comorbidities, polypharmacy, physical function measures and antidepressant medication use at baseline. Adjusting for these factors, the risk of dying or developing dementia or persistent physical disability during the follow-up period was 50% higher for women with LLD compared with women with minimal or no depressive symptoms. In male participants, the association between LLD and lower disability-free survival was significant adjusting for sociodemographic and lifestyle factors but became non-significant after adjustment was made for medical comorbidities and polypharmacy. Subthreshold depressive symptoms were also associated with lower disability-free survival in both sexes. The magnitudes of these associations were smaller than those for LLD, suggesting the existence of a dose-response relationship regarding the effect of depressive symptoms on disability-free survival. Taken together, these findings suggest that LLD may have a causal, and not merely associational relationship, with lower disability-free survival.

The study also partially replicated previous findings that LLD is prospectively associated with all-cause mortality, dementia and physical disability.^8,10,11^ In female participants, LLD was associated with both all-cause mortality and dementia adjusting for all covariates. In male participants, subthreshold depression was associated with all-cause mortality adjusting for age and race. In both sexes, LLD was associated with persistent physical disability. This was by far the strongest relationship that existed between LLD and the endpoints. Adjusting for all covariates, the risk of developing persistent physical disability during the follow-up period was 87% higher for female participants with LLD compared with those with minimal or no depressive symptoms.

The associations between depressive symptoms and the endpoints were in almost all cases stronger for female participants than male participants. This was unanticipated because previous studies have not generally found that LLD has more severe physical health impacts in females. It is possible that males’ shorter life expectancy and earlier onset of age-related morbidity meant that the inclusion criteria, which required participants to be free of physical disability and cardiovascular disease, resulted in a cohort of male participants who were healthier and therefore less susceptible to the effects of LLD than their female counterparts. The fact that female participants had a higher rate of polypharmacy at baseline than male participants (23.6% vs. 15.6%) lends some support to this possibility. Alternatively, the disparity may have been due to the study’s greater statistical power for women. The numbers (percentages) of female participants in the LLD and subthreshold depression groups were 1248 (11.6%) and 4378 (40.6%), respectively, compared with 631 (7.6%) and 2981 (35.9%) male participants. However, the widths of the confidence intervals for most endpoints were similar for both sexes, indicating that statistical power is at most only part of the explanation.

There are likely multiple biopsychosocial mechanisms underlying the associations found between LLD and the endpoints. Depression has a complex and bidirectional relationship with physical illness and numerous biological, behavioural and psychosocial mechanisms are believed to contribute to this relationship. One intriguing but speculative hypothesis is that depression and other psychiatric disorders involve common pathophysiological processes that contribute concurrently to the development of physical morbidity. Such processes may include the activation of immune/inflammatory pathways, increased oxidative and nitrosative stress and mitochondrial dysfunction.^27^

Given the strong relationship that was found to exist between LLD and persistent physical disability, it is interesting to consider the mechanisms underlying this relationship. Little is known regarding these mechanisms, although proposed causal pathways include: (i) amotivation causing physical inactivity, leading to deconditioning and frailty, (ii) decreased appetite and poor nutrition contributing to skeletal muscle loss and sarcopenia, (iii) self-neglect leading to risky health behaviours such as alcohol abuse and thereby causing medical comorbidities that lead to disability and (iv) depression-related deficits in executive function impairing a depressed person’s ability to perform the cognitive aspects of ADLs.^11^ The results of this study suggest that these mechanisms may indeed mediate part of the relationship between LLD and physical disability. For example, the HRs for the endpoint of persistent physical disability decreased from 2.22 to 1.97 for women and from 2.17 to 1.75 for men after adjustment was made for physical function measures. This is consistent with factors such as inactivity, frailty and skeletal muscle loss partly mediating the relationship between LLD and physical disability. However, the fact that a strong relationship persisted even after adjustment for these measures suggests that there are other important mechanisms contributing to the relationship.

One possibility is that psychological mechanisms are operative. A psychological variable that may partially mediate the relationship between LLD and physical disability is apathy. Apathy is a common symptom of LLD, occurring in nearly 40% of cases.^28^ There is evidence that it is related to physical disability independently of other depressive symptoms.^28^ Apathy also appears to be associated with a decline in self-report measures of physical function but not in objective measures such as gait speed or handgrip strength.^29^ It is possible that apathy affects depressed older adults’ perceptions of their functional capacities and thereby contributes to an impairment of these capacities. If psychological variables partially mediate the relationship between LLD and physical disability, this suggests that depression-related disability, even if persistent, may be amenable to treatment.

This study has a number of strengths, including its large sample size, its prospective design, the use of a validated measure of depressive symptoms and the collection of data according to a well-designed randomised controlled trial protocol with rigorous procedures for assessing endpoints. Rates of attrition and missing data were low. The study also has some limitations. First, the CES-D-10 is a screening instrument and is not equivalent to diagnosis by a trained clinician using a semi-structured diagnostic instrument, the gold standard for psychiatric diagnosis. Second, a single cross-sectional CES-D-10 score was used to assign participants to the three groups. This may have inflated the LLD and subthreshold depression groups by, for instance, including participants experiencing transient mood symptoms in these groups. Third, the median follow-up period for the primary composite endpoint was only 4.7 and 4.5 years for female and male participants, respectively. This means that associations between LLD and lower healthspan could have been due to undiagnosed subclinical or prodromal conditions causing depressive symptoms. For instance, there is some evidence that prodromal dementia can cause depressive symptoms.^30^ Fourth, the study did not adjust for all potential confounders. For example, low subjective social status and physical pain are risk factors for LLD that were not controlled for.^31^ This is also consistent with LLD being merely a marker of risk factors for lower healthspan and not itself having any causal effects on healthspan. Finally, study participants were healthier than the overall population of older adults, which may limit generalisability.

## 5 | CONCLUSION

This study suggests that LLD may be a common risk factor for shortened healthspan. This underscores the importance of identifying and treating depression for healthy ageing.

## DATA AVAILABILITY STATEMENT

Research data are not shared.

## Supplementary Material

S1

S2

S3

S4

## Figures and Tables

**TABLE 1:** Baseline sociodemographic and lifestyle characteristics, rates of common medical conditions and polypharmacy, performance on physical function measures, rates of antidepressant medication use and mean CES-D-10 scores for late-life depression, subthreshold depression and no depression groups

	Female participants			Male participants		
Variable	No depression *n* (%)^a^	Subthreshold depression *n* (%)^a^	LLD *n* (%)^a^	No depression *n* (%)^a^	Subthreshold depression *n* (%)^a^	LLD *n* (%)^a^
Total *n* (%)	5153 (47.8%)*	4378 (40.6%)*	1248 (11.6%)*	4719 (56.8%)*	2981 (35.9%)*	631 (7.6%)*
Age M (*SD*)	75.1 (4.6)*	75.4 (4.6)*	75.2 (4.6)*	74.9 (4.4)	75.0 (4.5)	75.1 (4.8)
Race						
White/Caucasian	4756 (93.3%)*	4031 (93.1%)*	1115 (90.1%)*	4423 (94.6%)*	2797 (94.8%)*	572 (91.7%)*
Other	341 (6.7%)*	298 (6.9%)*	122 (9.9%)*	254 (5.4%)*	152 (5.2%)*	52 (8.3%)*
BMI (kg/m^2^) M (*SD*)	27.9 (5.1)*	28.3 (5.3)*	28.9 (5.8)*	27.9 (3.8)*	28.0 (4.1)*	28.3 (4.3)*
Alcohol use history						
Current	3743 (72.6%)*	3100 (70.8%)*	864 (69.2%)*	3912 (82.9%)*	2518 (84.5%)*	501 (79.4%)*
Former	210 (4.1%)*	238 (5.4%)*	81 (6.5%)*	328 (7.0%)*	206 (6.9%)*	74 (11.7%)*
Never	1200 (23.3%)*	1040 (23.8%)*	303 (24.3%)*	479 (10.2%)*	258 (8.7%)*	56 (8.9%)*
Smoking history						
Current	149 (2.9%)*	144 (3.3%)*	58 (4.6%)*	191 (4.0%)*	140 (4.7%)*	52 (8.2%)*
Former	1563 (30.3%)*	1437 (32.8%)*	436 (34.9%)*	2418 (51.2%)*	1623 (54.4%)*	320 (50.7%)*
Never	3441 (66.8%)*	2797 (63.9%)*	754 (60.4%)*	2110 (44.7%)*	1218 (40.9%)*	259 (41.0%)*
Accommodation status						
At home with another person	3112 (60.4%)*	2449 (55.9%)*	645 (51.7%)*	3857 (81.7%)*	2296 (77.0%)*	418 (66.2%)*
At home alone or in residential care	2041 (39.6%)*	1929 (44.1%)*	603 (48.3%)*	862 (18.3%)*	685 (23.0%)*	213 (33.8%)*
Level of education						
≤12 yrs education	2942 (57.1%)*	2632 (60.1%)*	778 (62.4%)*	2583 (54.7%)	1643 (55.1%)	373 (59.1%)
>12 yrs education	2211 (42.9%)*	1746 (39.9%)*	469 (37.6%)*	2136 (45.3%)	1338 (44.9%)	258 (40.9%)
Medical conditions						
Hypertension	3777 (73.3%)*	3286 (75.1%)*	953 (76.4%)*	3582 (75.9%)	2214 (74.3%)	482 (76.4%)
Dyslipidaemia	3711 (72.5%)	3112 (71.6%)	898 (72.3%)	2532 (54.1%)	1599 (54.0%)	338 (53.7%)
Diabetes mellitus	429 (8.3%)*	425 (9.7%)*	145 (11.6%)*	542 (11.5%)*	398 (13.4%)*	105 (16.6%)*
CKD	214 (4.2%)*	228 (5.2%)*	64 (5.1%)*	888 (18.8%)*	590 (19.8%)*	153 (24.2%)*
Respiratory disease	731 (14.2%)*	697 (15.9%)*	228 (18.3%)*	566 (12.0%)*	448 (15.0%)*	111 (17.6%)*
GORD	1354 (26.3%)*	1495 (34.1%)*	461 (36.9%)*	1152 (24.4%)*	855 (28.7%)*	220 (34.9%)*
Gout	97 (1.9%)*	111 (2.5%)*	53 (4.2%)*	524 (11.1%)	332 (11.1%)	72 (11.4%)
Parkinson’s disease	39 (0.8%)*	53 (1.2%)*	31 (2.5%)*	37 (0.8%)*	50 (1.7%)*	16 (2.5%)*
Cancer history	871 (17.0%)	758 (17.4%)	241 (19.4%)	990 (21.1%)	648 (21.8%)	152 (24.3%)
Polypharmacy	994 (19.3%)*	1153 (26.3%)*	397 (31.8%)*	616 (13.1%)*	525 (17.6%)*	156 (24.7%)*
Grip strength M (*SD*) (kg)	21.0 (5.8)*	20.4 (5.8)*	20.0 (6.0)*	35.2 (8.4)*	34.7 (8.4)*	33.9 (9.1)*
Time to walk 3 m M (*SD*) (s)	3.2 (0.9)*	3.3 (1.1)*	3.5 (1.2)*	3.0 (0.7)*	3.0 (0.8)*	3.2 (1.1)*
Longest time walking without rest M (*SD*) (mins)						
<10 minutes	344 (6.7%)*	408 (9.3%)*	165 (13.2%)*	226 (4.8%)*	202 (6.8%)*	57 (9.1%)*
10–15 minutes	449 (8.7%)*	489 (11.2%)*	146 (11.7%)*	359 (7.6%)*	278 (9.4%)*	73 (11.6%)*
16–30 minutes	1129 (21.9%)*	1042 (23.9%)*	316 (25.4%)*	826 (17.5%)*	649 (21.8%)*	170 (27.0%)*
>30 minutes	3222 (62.6%)*	2428 (55.6%)*	619 (49.7%)*	3298 (70.0%)*	1844 (62.0%)*	329 (52.3%)*
Use of antidepressant medications	542 (10.5%)*	667 (15.2%)*	342 (27.4%)*	225 (4.8%)*	254 (8.5%)*	114 (18.1%)
CES-D-10 score M (*SD*)	0.9 (0.8)*	4.5 (1.3)*	10.7 (3.2)*	0.8 (0.8)*	4.4 (1.3)*	10.5 (3.0)*

*Note*: The three groups were defined according to baseline CES-D-10 score: no depression (CES-D-10 score ≤2), subthreshold depression (CES-D-10 score 3–7) and LLD (CES-D-10 score ≥8). Medical conditions were defined according to the criteria in Table S1 in the Supplementary Materials to.^26^ These criteria were based on self-reported history of these conditions, use of medications to treat them, participants’ vital signs and blood and urine test results. Because an expanded questionnaire concerning participants’ medical history was introduced in June 2013, self-report data were missing for 12,108 participants for respiratory disease, 10,747 participants for GORD, 12,505 participants for gout and 12,912 participants for Parkinson’s disease. Therefore, the definitions of these conditions were for most participants based upon the use of medications to treat them. Polypharmacy was defined as the simultaneous use of ≥5 medications. Grip strength was defined as the mean grip strength in the dominant hand after three trials. Gait speed was defined as the mean time to walk 3 m after two trials. Longest time walking without rest was defined as the longest time that participants reported walking without sitting down to rest over the previous 2 weeks. Data were missing for 197 participants for race, 89 participants for BMI, 1 participant for education status, 133 participants for dyslipidaemia and 42 participants for longest walking time.

Abbreviations: BMI, body mass index; CES-D-10, Centre for Epidemiological Studies Depression Scale 10-item version; CKD, chronic kidney disease; GORD, gastroesophageal reflux disease; LLD, late-life depression; *M*, mean; *SD*, standard deviation.

a Except where indicated.

* *p* < 0.05.

**TABLE 2:** Incidences of primary composite endpoint and secondary endpoints (all-cause mortality, incident dementia and incident persistent physical disability) in late-life depression, subthreshold depression and no depression groups during follow-up period

	Female participants				Male participants			
Endpoint	Total number of events during follow-up	Total follow-up period (person-years)	Incidence (events per 1000 person-years)	95% CI	Total number of events during follow-up	Total follow-up period (person-years)	Incidence (events per 1000 person-years)	95% CI
*Primary composite endpoint*								
No depression	365	23,610	15	14–17	478	21,277	22	20–25
Subthreshold depression	397	19,913	20	18–22	351	13,112	27	24–30
LLD	156	5437	29	24–34	88	2665	33	26–41
*All-cause mortality*								
No depression	184	24,179	8	7–9	309	21,767	14	13–16
Subthreshold depression	211	20,492	10	9–12	225	13,483	17	15–19
LLD	74	5682	13	10–16	49	2768	18	13–23
*Dementia*								
No depression	130	23,279	6	5–7	156	20,971	7	6–9
Subthreshold depression	124	19,602	6	5–8	92	12,909	7	6–9
LLD	48	5327	9	7–12	25	2621	10	6–14
*Persistent physical disability*								
No depression	83	21,165	4	3–5	72	19,179	4	3–5
Subthreshold depression	102	17,656	6	5–7	79	11,717	7	5–8
LLD	53	4737	11	8–15	23	2365	10	6–15

*Note*: The primary composite endpoint was assessed as the first event of death, dementia or persistent physical disability.

Abbreviations: CI, confidence interval; LLD, late-life depression.

**TABLE 3:** Results of Cox proportional hazards regression analysis investigating late-life depression and subthreshold depression as predictors of primary composite endpoint and secondary endpoints (all-cause mortality, incident dementia and incident persistent physical disability) in Models 1 to 5

	Model 1		Model 2		Model 3		Model 4		Model 5		
Endpoint	HR	95% CI	HR	95% CI	HR	95% CI	HR	95% CI	HR	95% CI	p-value
**Primary composite endpoint**											
*Female participants*											
Subthreshold depression	1.24	1.08–1.43	1.22	1.05–1.40	1.19	1.03–1.38	1.15	0.99–1.33	1.14	0.98–1.32	<0.001*^a^
LLD	1.87	1.55–2.25	1.79	1.48–2.16	1.72	1.42–2.08	1.54	1.26–1.87	1.50	1.23–1.82	
*Male participants*											
Subthreshold depression	1.22	1.06–1.40	1.19	1.03–1.36	1.13	0.98–1.30	1.08	0.93–1.24	1.06	0.92–1.22	0.746
LLD	1.39	1.11–1.75	1.30	1.03–1.64	1.25	0.99–1.59	1.09	0.86–1.38	1.04	0.82–1.32	
**All-cause mortality**											
*Female participants*											
Subthreshold depression	1.29	1.05–1.57	1.26	1.03–1.54	1.26	1.03–1.55	1.22	1.00–1.50	1.22	0.99–1.49	0.029*
LLD	1.71	1.30–2.24	1.63	1.24–2.14	1.63	1.23–2.14	1.49	1.13–1.98	1.44	1.08–1.91	
*Male participants*											
Subthreshold depression	1.19	1.01–1.42	1.16	0.97–1.38	1.08	0.90–1.28	1.05	0.88–1.26	1.04	0.87–1.24	0.501
LLD	1.16	0.85–1.57	1.05	0.77–1.43	0.99	0.72–1.35	0.88	0.64–1.22	0.86	0.62–1.18	
**Dementia**											
*Female participants*											
Subthreshold depression	1.10	0.86–1.40	1.09	0.85–1.40	1.08	0.84–1.38	1.06	0.82–1.36	1.06	0.82–1.36	0.147
LLD	1.65	1.18–2.30	1.66	1.19–2.31	1.60	1.14–2.24	1.45	1.02–2.05	1.43	1.01–2.03	
*Male participants*											
Subthreshold depression	0.98	0.76–1.27	0.99	0.76–1.28	0.96	0. 74–1.25	0.90	0.69–1.18	0.88	0.67–1.15	0.562
LLD	1.28	0.84–1.96	1.23	0.80–1.88	1.26	0.82–1.94	1.13	0.73–1.75	1.06	0.68–1.66	
**Persistent physical disability**											
*Female participants*											
Subthreshold depression	1.44	1.08–1.92	1.35	1.01–1.81	1.25	0.93–1.67	1.22	0.90–1.66	1.20	0.88–1.63	0.006*^a^
LLD	2.87	2.03–4.06	2.52	1.78–3.58	2.22	1.56–3.18	1.97	1.35–2.85	1.87	1.29–2.73	
*Male participants*											
Subthreshold depression	1.86	1.35–2.56	1.80	1.30–2.49	1.68	1.21–2.33	1.52	1.08–2.12	1.47	1.05–2.06	0.045*
LLD	2.52	1.56–4.06	2.43	1.49–3.95	2.17	1.33–3.54	1.75	1.06–2.90	1.61	0.97–2.68	

*Note*: The primary composite endpoint was assessed as the first event of death, dementia or persistent physical disability. The reference class is the no depression group. Model 1 adjusted for sociodemographic factors (age and race). Model 2 further adjusted for lifestyle factors (BMI, alcohol use history, smoking history, level of education and accommodation status). Model 3 further adjusted for the presence of common medical conditions (hypertension, dyslipidaemia, diabetes mellitus, CKD, respiratory disease, GORD, gout, Parkinson’s disease and a cancer history) and polypharmacy. Model 4 further adjusted for measures of physical function (grip strength, gait speed and longest time walking without rest). Model 5 further adjusted for use of antidepressant medications at baseline. An *F*-test of overall significance was performed to calculate *p*-values for the three-way comparisons for each endpoint in Model 5.

Abbreviations: CI, confidence interval; HR, hazard ratio; LLD, late-life depression.

a Statistically significant according to the Benjamini–Hochberg procedure with the false discovery rate set to 0.05.

* *p* < 0.05.

## Significant outcomes

- This study found that late-life depression was associated with lower disability-free survival in physically healthy older women adjusting for a wide range of potential confounders, including sociodemographic and lifestyle factors, medical comorbidities, polypharmacy, physical function and antidepressant use.
- It also found evidence of a dose-response relationship between depressive symptoms and disability-free survival.
- These findings suggest that late-life depression may be a common and important risk factor for shortened healthy life span, highlighting the importance of identifying and treating this condition.

## Limitations

- A screening instrument was used to measure depressive symptoms and assign participants to the late-life depression group rather than assessment by a trained clinician using a semi-structured diagnostic instrument, the gold standard for psychiatric diagnosis.
- Depressive symptoms were assessed cross-sectionally, which may have meant that participants experiencing transient mood symptoms were included in the late-life depression group.
- The study did not adjust for all potential confounders and therefore cannot exclude the possibility that the relationship between late-life depression and lower disability-free survival was associational and not causal.
