# Supplementary figures and images for: The effect of depressive symptoms on disability-free survival in healthy older adults: A prospective cohort study
Source: Acta Psychiatr Scand. Author manuscript; Available in PMC 2024 Jan 1. (PMC10026010; doi:10.1111/acps.13513)

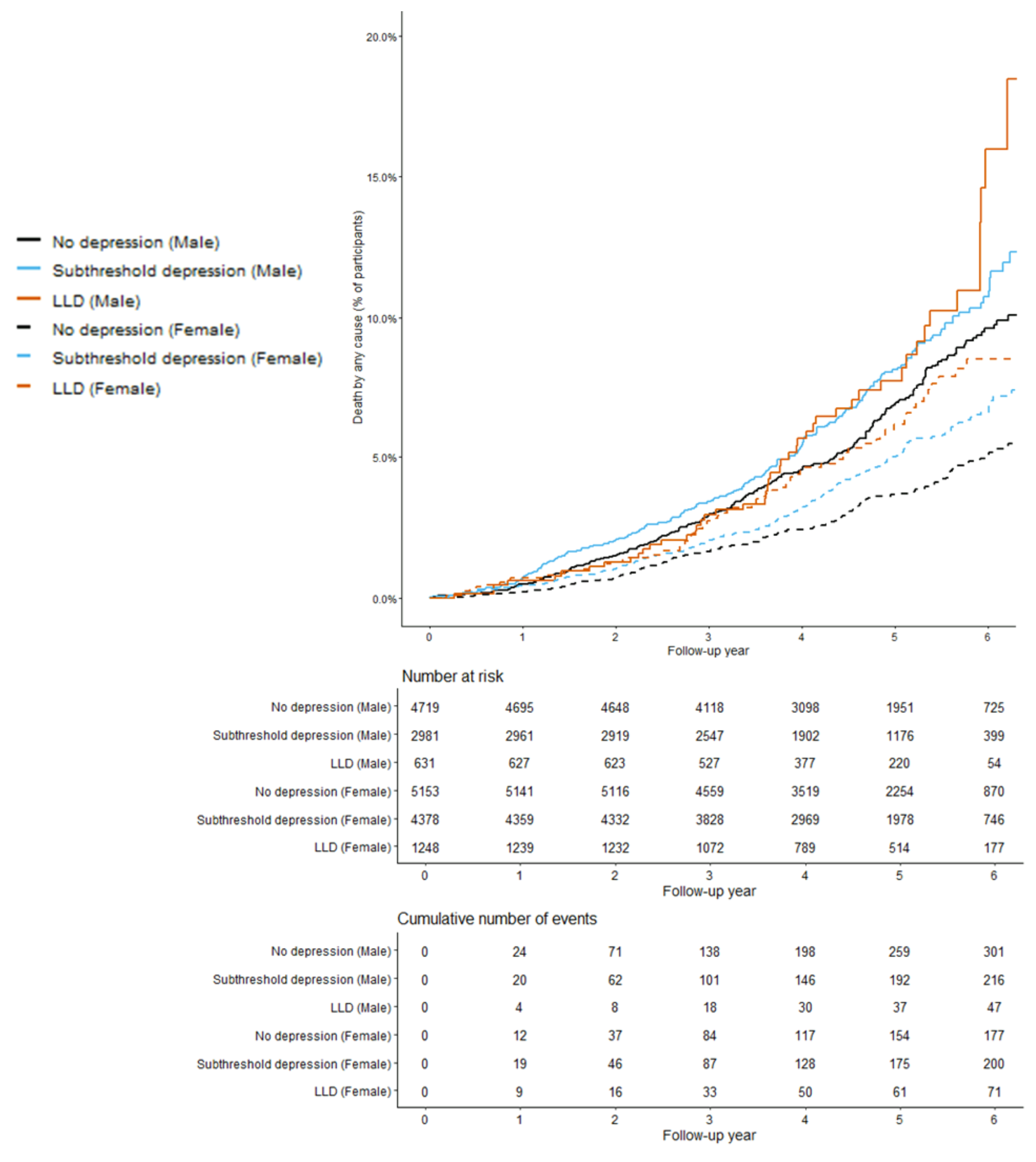

Supplement: S1 [file NIHMS1878680-supplement-S1.png]

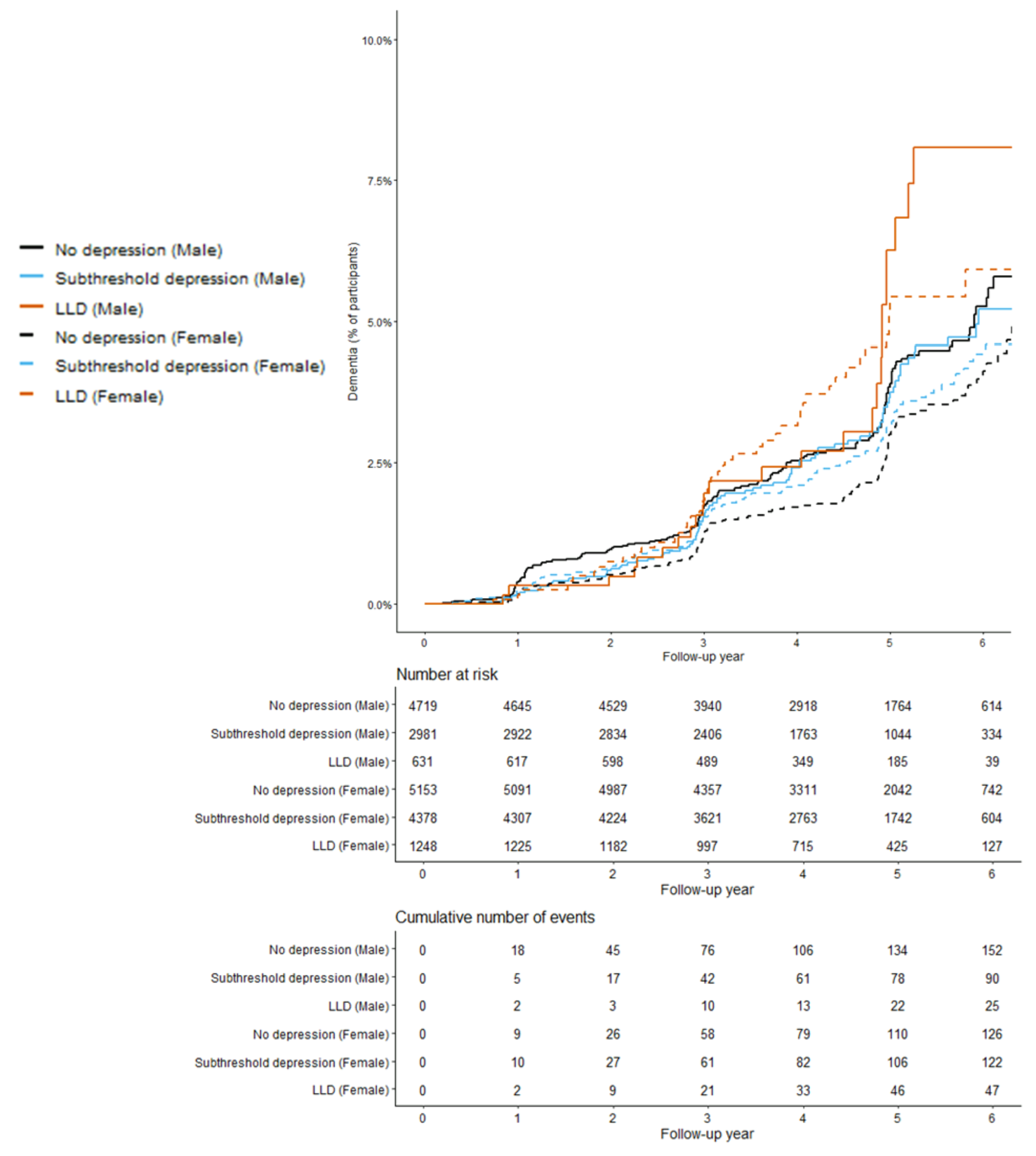

Supplement: S2 [file NIHMS1878680-supplement-S2.png]

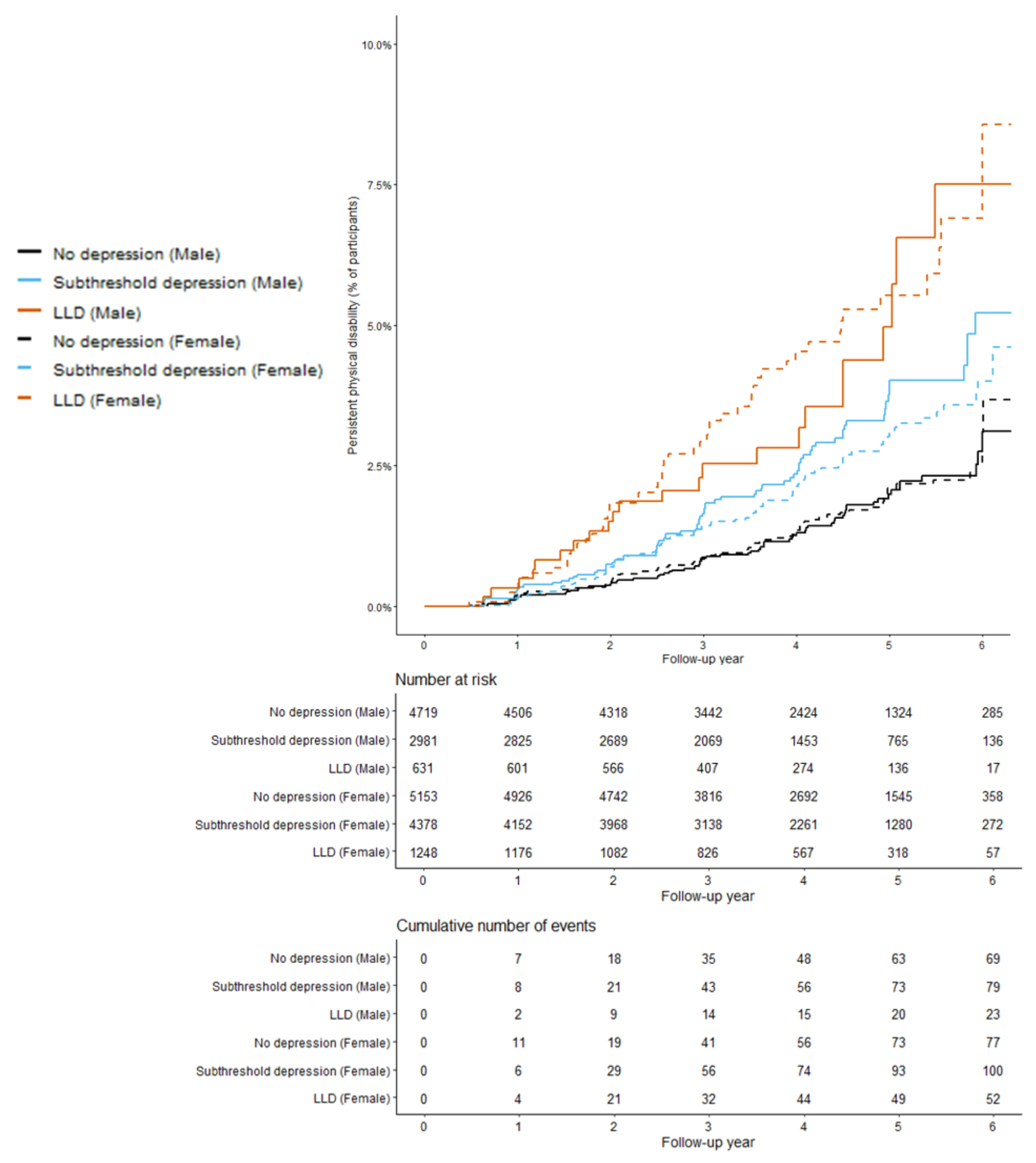

Supplement: S3 [file NIHMS1878680-supplement-S3.png]
