# Supplementary material for: The effect of depressive symptoms on disability-free survival in healthy older adults: A prospective cohort study
Source: Acta Psychiatr Scand. Author manuscript; Available in PMC 2024 Jan 1. (PMC10026010; doi:10.1111/acps.13513)
Supplement: S4 [file NIHMS1878680-supplement-S4.docx]

**6. Supplementary materials**

**Table S1. Results of Cox proportional hazards regression analysis investigating predictors of primary composite endpoint in Model 5**

| **Variable** | **Women** | | **Men** | |  |
| --- | --- | --- | --- | --- | --- |
|  | **HR** | **95% CI** | **HR** | **95% CI** |  |
| Age | 1.10 | 1.09-1.12 | 1.09 | 1.08-1.11 |  |
| Race: other**†** | 0.68 | 0.49-0.93 | 1.10 | 0.81-1.51 |  |
| BMI | 1.00 | 0.99-1.01 | 0.98 | 0.96-1.00 |  |
| Alcohol use history**†** |  |  |  |  |  |
| Former | 1.65 | 1.27-2.15 | 1.10 | 0.87-1.40 |  |
| Never | 1.27 | 1.09-1.49 | 1.18 | 0.94-1.47 |  |
| Smoking history**†** |  |  |  |  |  |
| Former | 0.65 | 0.46-0.90 | 0.55 | 0.42-0.71 |  |
| Never | 0.56 | 0.40-0.78 | 0.46 | 0.35-0.60 |  |
| Accommodation status**†** | 0.93 | 0.81-1.06 | 0.88 | 0.75-1.03 |  |
| Level of education**†** | 0.93 | 0.81-1.07 | 0.85 | 0.74-0.98 |  |
| Hypertension | 1.03 | 0.87-1.22 | 1.07 | 0.91-1.27 |  |
| Dyslipidaemia | 1.03 | 0.89-1.20 | 0.93 | 0.81-1.07 |  |
| Diabetes mellitus | 1.30 | 1.05-1.60 | 1.23 | 1.01-1.49 |  |
| CKD | 0.85 | 0.62-1.15 | 1.20 | 1.02-1.41 |  |
| Respiratory disease | 1.17 | 0.97-1.42 | 1.33 | 1.10-1.61 |  |
| GORD | 1.08 | 0.93-1.26 | 0.91 | 0.78-1.07 |  |
| Gout | 1.34 | 0.91-1.96 | 0.80 | 0.63-1.02 |  |
| Parkinson’s disease | 1.78 | 1.12-2.83 | 2.90 | 1.95-4.31 |  |
| Cancer history | 1.22 | 1.04-1.43 | 1.23 | 1.05-1.43 |  |
| Polypharmacy | 0.91 | 0.77-1.08 | 0.88 | 0.72-1.07 |  |
| Grip strength | 0.98 | 0.97-0.99 | 0.98 | 0.97-0.99 |  |
| Time to walk 3 m | 1.30 | 1.24-1.36 | 1.17 | 1.10-1.25 |  |
| Longest time walking without rest | 0.90 | 0.84-0.96 | 0.84 | 0.79-0.90 |  |
| Use of antidepressant medications at baseline**†** | 1.22 | 1.02-1.47 | 1.58 | 1.26-1.98 |  |
| Subthreshold depression**†** | 1.14 | 0.98-1.32 | 1.06 | 0.92-1.22 |  |
| LLD**†** | 1.50 | 1.23-1.82 | 1.04 | 0.82-1.32 |  |
| *Abbreviations*: LLD, late-life depression; BMI, body mass index; CKD, chronic kidney disease; GORD, gastroesophageal reflux disease; CES-D-10, Center for Epidemiological Studies Depression Scale 10-item version.  †The relevant reference classes for these variables are White/Caucasian race, current alcohol use, current smoker, living alone, education ≤12 years, not using antidepressant medications at baseline and no depression. | | | | | |

**Figure S1. Cumulative incidence of death by any cause for the three groups for female and male participants**

**Figure S2. Cumulative incidence of dementia for the three groups for female and male participants**

**Figure S3. Cumulative incidence of persistent physical disability for the three groups for female and male participants**
